# Protonated paramagnetic redox forms of di-*o*-quinone bridged with *p*-phenylene-extended TTF: A EPR spectroscopy study

**DOI:** 10.3762/bjoc.12.238

**Published:** 2016-11-17

**Authors:** Nikolay O Chalkov, Vladimir K Cherkasov, Gleb A Abakumov, Andrey G Starikov, Viacheslav A Kuropatov

**Affiliations:** 1 Laboratory of Organoelement Compounds, G.A. Razuvaev Institute of Organometallic Chemistry of RAS, 603950, GSP-445, Tropinina str., 49, Nizhny Novgorod, Russia; 2 N. I. Lobachevsky Nizhny Novgorod State University, Gagarina av., 23, Nizhny Novgorod, Russia; 3 Southern Scientific Center of Russian Academy of Science, 344006, Chekhov str., 41, Rostov-on-Don, Russia

**Keywords:** acceptor–donor–acceptor triad, bridging ligand, EPR spectroscopy, extended tetrathiafulvalene, protonated semiquinone

## Abstract

The chemical oxidation and reduction processes of deprotonated, direduced *o*-quinone-exTTF-*o*-quinone in protic solvents were studied by EPR spectroscopy. The formation of relatively stable paramagnetic protonated redox forms of the parent triad was very surprising. The character of spin-density distribution in the semiquinone–quinone and semiquinone–catechol redox forms indicates that the *p*-phenylene-extended tetrathiafulvalene connector provides a quite effective electronic communication channel between dioxolene coordination sites. It was found that the deprotonated, direduced *o*-quinone-exTTF-*o*-quinone is capable to reduction of the metal copper in solution. The radical anion species formed in this reaction exists in solution as a solvent-separated ion pair with a copper cation. A character of spin-density distribution in a radical anion species leads to the conclusion that the ligand corresponds to type III of the Robin–Day classification.

## Introduction

**Figure 1:**
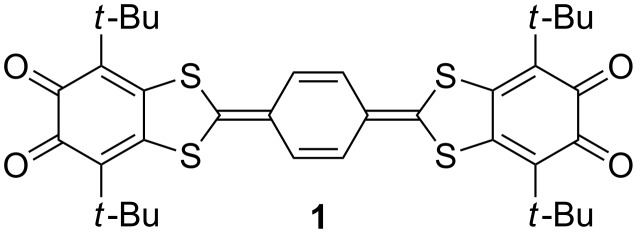
The structural formula of acceptor–donor–acceptor triad **1**.

The main idea that led to the creation of the system constructed of two *o*-quinone terminal moieties bridged with annulated extended tetrathiafulvalene (TTF) insertion, was an attempt to explore acceptor–donor–acceptor (A–D–A) systems as ligands [1]. A linear planar skeleton of the molecule with coordinating sites placed at the termini allows the construction of ordered structures using metal ions as nodes [2]. The insertion of *p*-phenylene in the TTF core is of special interest because it can act as a switch which drives electronic communication between paramagnetic centers at the termini. Coordination compounds and other derivatives containing the quinone-exTTF-quinone system in a paramagnetic state are numerous, and EPR spectroscopy seems best suited for the study of such objects. The coupling constants with protons of the central *p*-phenylene ring are highly dependent on the geometry of the ligand as well as on the coordination surrounding at their terminal dioxolene sites. Thus the EPR spectrum contains plenty of information about the structure of the molecule as well as about the dynamic processes proceeding at the coordination sites. Previously we reported paramagnetic derivatives of di-*o*-quinone (**1**, Figure 1) with alkali metals [3]. Using the protonated paramagnetic derivatives of the quinone-exTTF-quinone system we succeeded in organization of a symmetric surrounding for both coordination sites of the molecule. This allowed us to estimate the contribution of exTTF insertion to the mechanism of the electronic communication between the chelating centers of the molecule.

## Results and Discussion

Despite almost all protonated *o*-semiquinones being very labile species, the direduced, diprotonated derivative of di-*o*-quinone **(1)H_2_** is stable in air both in the crystalline form and in solution. By means of chemical oxidation or reduction of **(1)H_2_** in a protic solvent it is possible to generate the corresponding protonated monoreduced or triprotonated trireduced forms, respectively. We studied the features of the spin-density distribution in the protonated paramagnetic redox forms of di-*o*-quinone **1**.

Open-shell DFT calculations performed for **(1)H_2_** at the B3LYP/6-311++G(d,p) level of theory revealed a singlet biradical as a ground state. The value of antiferromagnetic coupling was estimated as 1092 cm^−1^ [1]. Due to this, the species **(1)H_2_** is EPR silent and moreover, it exhibits narrow peaks in the ^1^H and ^13^C NMR spectra.

**Figure 2:**
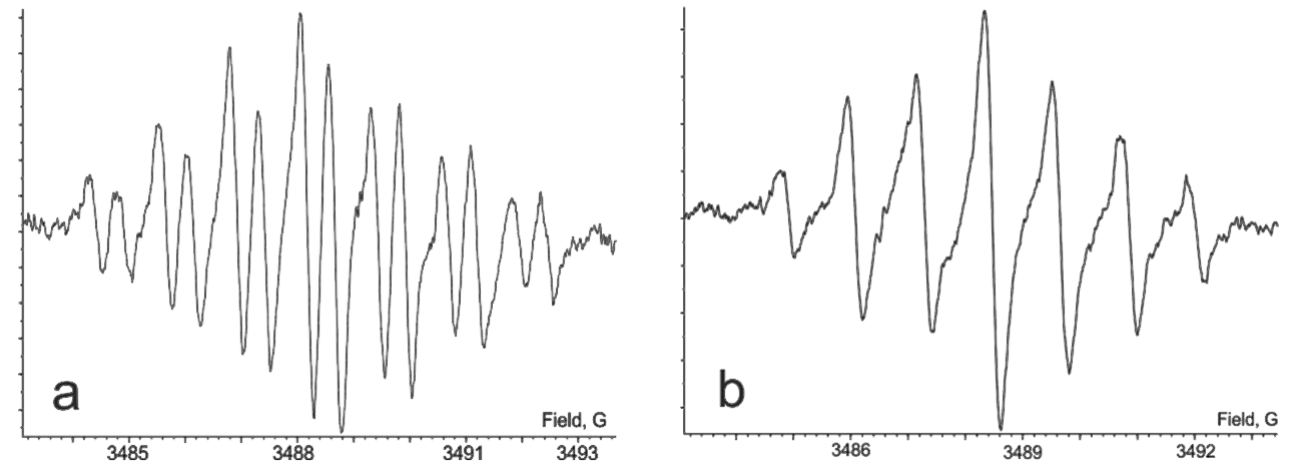
The EPR spectrum of **(1^·^)H** in CHCl_3_, 293 K: a) experimental and b) experimental + D_2_O.

**Scheme 1:**
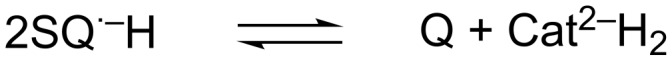
Disproportionation of the protonated semiquinones in solution.

We previously reported that chemical oxidation of **(1)H_2_** with lead(IV) oxide in solution results in the quantitative formation of the di-*o*-quinone form **1**. The EPR spectroscopical monitoring of this reaction in chloroform allowed us to observe a multiplet signal centered at *g*_iso_ = 2.0051 which corresponds to an intermediate paramagnetic species. The resulting spectrum (Figure 2a) is a triplet (1:2:1) of triplets (1:2:1) of doublets and it was attributed to the protonated semiquinonate radical anion. The integral intensity of the spectrum is relatively high. This is an unusual phenomenon, since typically protonated semiquinones tend to disproportionate to the corresponding catecholate and *o*-quinone (Scheme 1). The equilibrium in this process commonly lies in favor of the diamagnetic products [4–6]. This leads to the fact that the EPR signal related to a protonated semiquinone species is often quite weak or even undetectable. In the spectrum, two separate triplet splittings of 2.52 and 1.26 G are attributable to the hyperfine interaction with protons of the central *p*-phenylene ring. The doublet splitting (0.49 G) arises from a magnetic interaction of an odd electron with a proton localized at the semiquinone coordination site. In order to corroborate this hypothesis we added a small amount of D_2_O to the solution of radical **(1^·^)H**. The only hydrogen which might be rapidly exchanged under such conditions is the proton attached to the semiquinone. Due to deuterium exchange the spectrum transforms into a broadened triplet of triplets (Figure 2b), since the higher spin (S = 3/2) and the small gyromagnetic ratio of the deuterium nucleus (γ_N_ = 4.1065 × 10^3^ rad G^−1^ s^−1^) renders the hyperfine constant too small to be resolved.

**Scheme 2:**
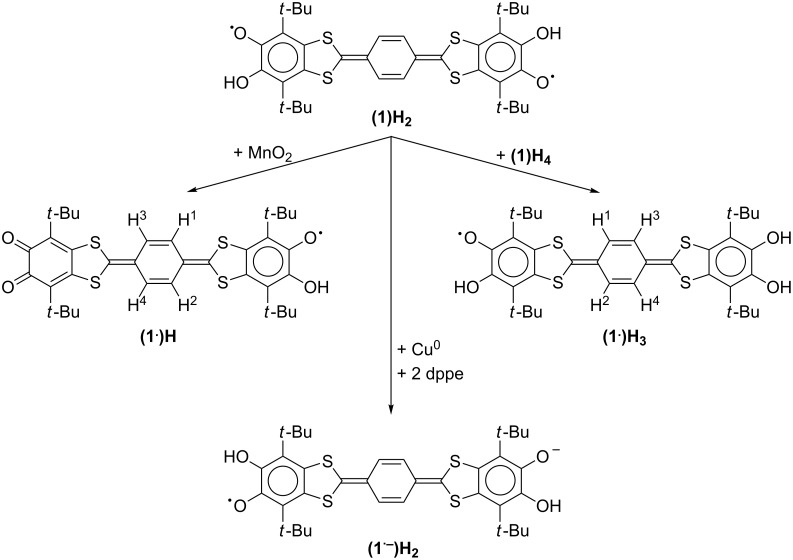
Paramagnetic reduced protonated derivatives of the quinone **2**.

There are two kinds of protons present in the central *p*-phenylene ring and it is a problem in attributing HFC constants to the specific protons. We performed DFT calculations at the UB3LYP/6-311++G(d,p) level of theory in order to estimate the spin-density distribution in the molecule of **(1^·^)H**. According to these data the observed hyperfine coupling constants were assigned to specific protons of the central *p*-phenylene ring: the larger splitting constant is ascribable to the H^3^ and H^4^ protons, whereas the smaller one corresponds to the H^1^ and H^2^ protons (see Scheme 2 and Supporting Information File 1).

The pairwise equivalence of H^1^–H^2^ and H^3^–H^4^ *p*-phenylene protons in the EPR spectrum originates from a prototropic tautomerism within a single dioxolene coordination site [7–8]. Meanwhile, the exchange of this semiquinone proton between the different coordination sites should cause an equalization of the proton constants on the *p*-phenylene group in the case of a fast process. In fact, we have not found any evidence of such migration, at least accessible for an observation in the EPR timescale. The lack of a substantial temperature dependence of the shape of lines in the EPR spectrum means that inhomogeneous broadening of the lines is absent.

**Figure 3:**
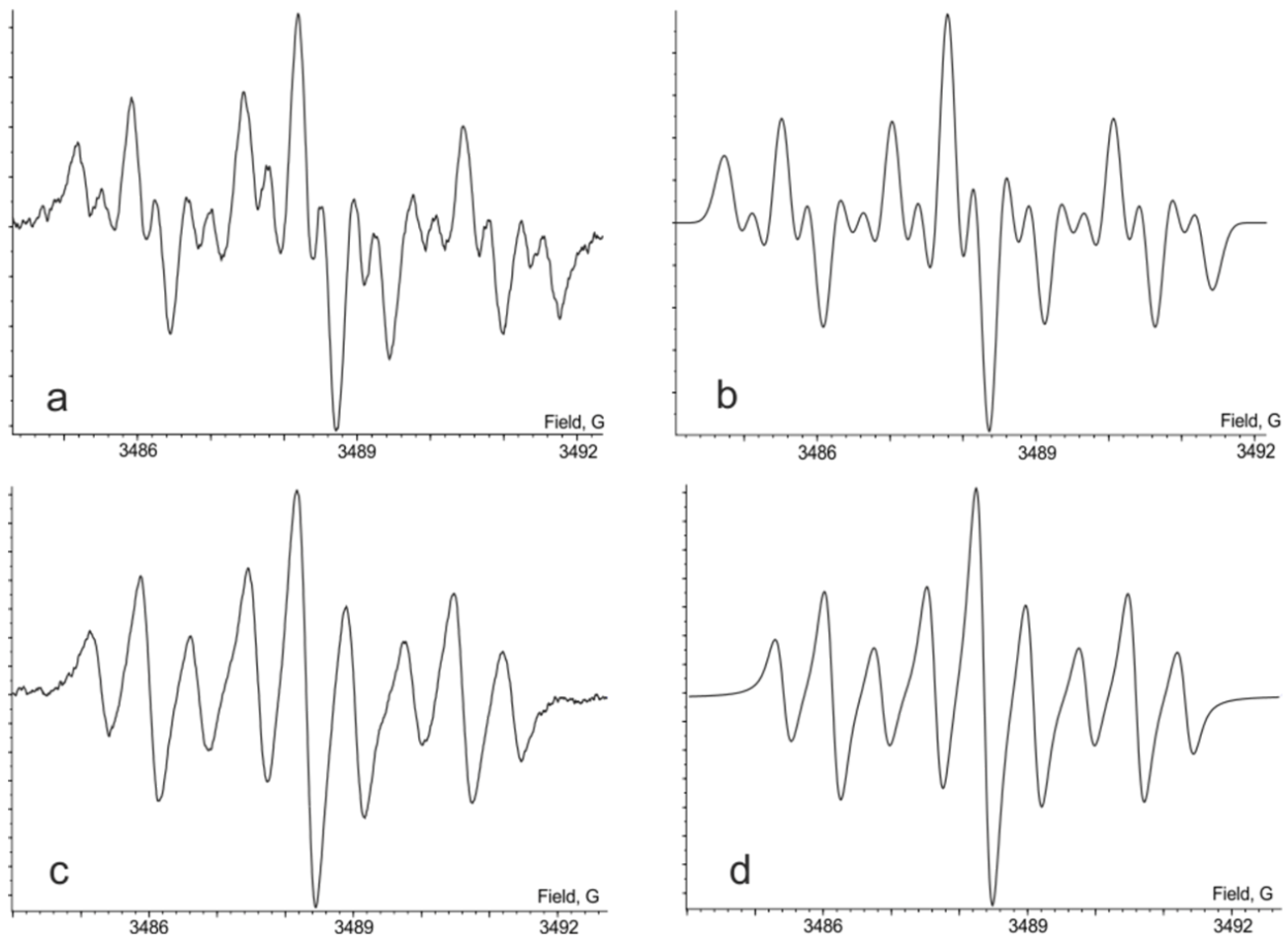
The EPR spectrum of **(1^·^)H_3_** in CHCl_3_, 293 K: a) experimental, b) simulated, c) experimental + D_2_O and d) simulated + D_2_O.

The triprotonated species **(1^·^)H_3_** was synthesized through the reduction of **(1)H_2_** with an equimolar amount of **(1)H_4_** in solution. The oxidation state of the quinone-exTTF-quinone core in **(1^·^)H_3_** corresponds to that in trianion radical metal complexes. The character of the spin-density distribution in this species is similar as it was observed for its monoprotonated analogue **(1^·^)H**. The EPR spectrum is centered at *g_iso_* = 2.0060 and it was also interpreted as a triplet of triplets (both 1:2:1) of doublets (Figure 3, Table 1). As it was observed in the case of **(1^·^)H** the addition of a small amount of D_2_O to the solution of **(1^·^)H_3_** in dichloromethane resulted in the disappearance of the doublet splitting from the spectrum. This means that doublet splitting arises due to magnetic interaction of an odd electron with a hydrogen atom localized at the semiquinone coordination site. The attribution of hyperfine coupling constants with specific protons was made according to data from DFT calculations. The similarity of characters of spin-density distribution in the corresponding mono- and trireduced species is an argument in favor of a similar geometry of the ligand in these derivatives. At the same time, when comparing **(1^·^)H** and **(1^·^)H_3_**, it is obvious that the presence of the catechol terminus in **(1^·^)H_3_** leads to a decrease in the acceptor ability of the whole ligand, and, in turn, results in smaller HFC constant values of the *p*-phenylene protons. The value of the hyperfine coupling constant of the catechol hydroxy protons (0.09 G) was estimated by means of a computer simulation of an experimental EPR spectrum, once the other hyperfine coupling constants had been identified.

**Table 1:** Hyperfine splitting constants and *g*-factors of protonated semiquinones. The values in brackets correspond to the analogous lithium derivatives.

Paramagnetic species, *g*-factor	H^1^, H^2^	H^3^,H^4^	OH

**(1^·^)H** 2.0051	1.26 (0.77)	2.50 (1.18)	0.49 (0.43)
**(1^·^)H_3_** 2.0060	0.74 (0.76)	2.28 (1.31)	0.30/0.09^a^ (0.41)^b^

^a^HFS constant of hydroxy protons on catechol terminus (estimation given by spectrum simulation). ^b^HFS constant of lithium nucleus.

The spin-density distribution in the protonated semiquinones **(1^·^)H** and **(1^·^)H_3_** substantially differs from that observed in their analogs with metal ions [3] (see Table 1). Tentatively, this feature could be related to a different bond ionicity of the semiquinone–metal and semiquinone–proton pairs, respectively.

The character of spin-density distributions in **(1^·^)H** and **(1^·^)H_3_** and the relatively high values of coupling constants with the protons in the EPR spectrum reveal that the *p*-phenylene-extended TTF bridge provides an effective electronic communication throughout the whole molecule. Obviously, the symmetry of the electron-density delocalization is largely determined by the configuration of the surrounding coordination sites at the termini of the molecule. Thus, **(1^.^)H** or **(1^.^)H_3_**, contain a protonated semiquinone at the one side and an *o*-quinone or catechol on the opposite side, respectively. As a result there is a non-symmetric spin-density distribution which puts obstacles in the way of understanding of the role of extended TTF insertion as an electronic exchange channel between the *o*-quinone coordination moieties.

The influence of *p*-phenylene insertion on the character of spin-density distribution is considerably easier to understand when the coordination environment at both termini of the molecule would be the same. Such situation is realized in the radical anion species **(1^·−.^)H_2_** which was generated during the reduction of **(1)H_2_** with metallic copper in the presence of diphenylphosphinoethane (dppe).

**Figure 4:**
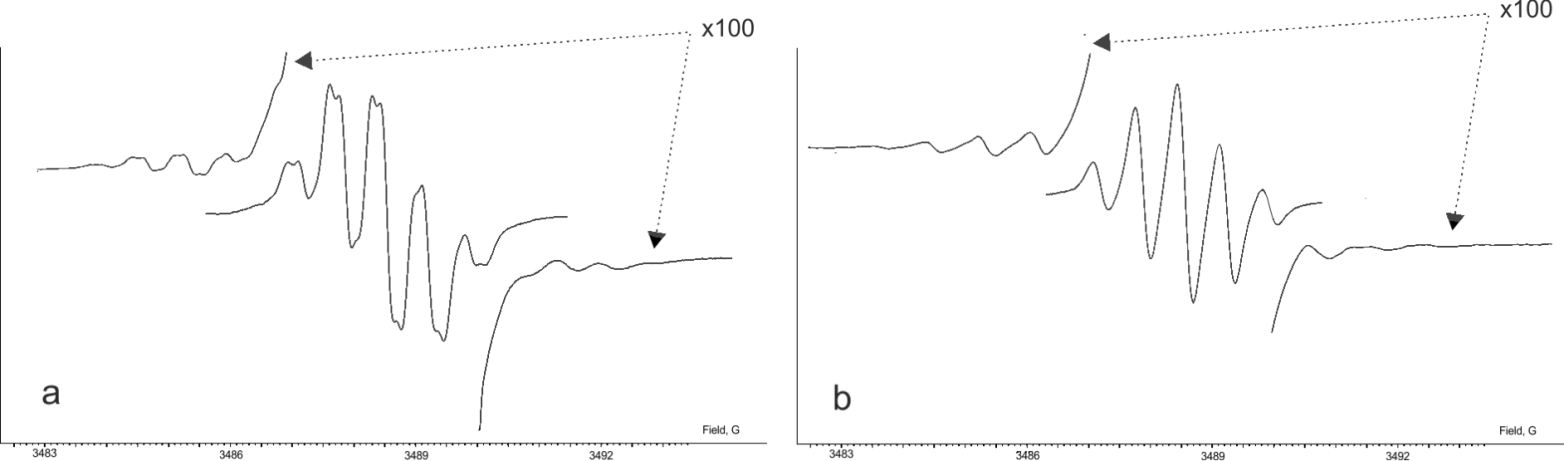
The EPR spectrum of **(1^·−^)H_2_** THF, 293 K: a) experimental and b) experimental + D_2_O). Magnified side lines correspond to ^33^S satellite components.

**Figure 5:**
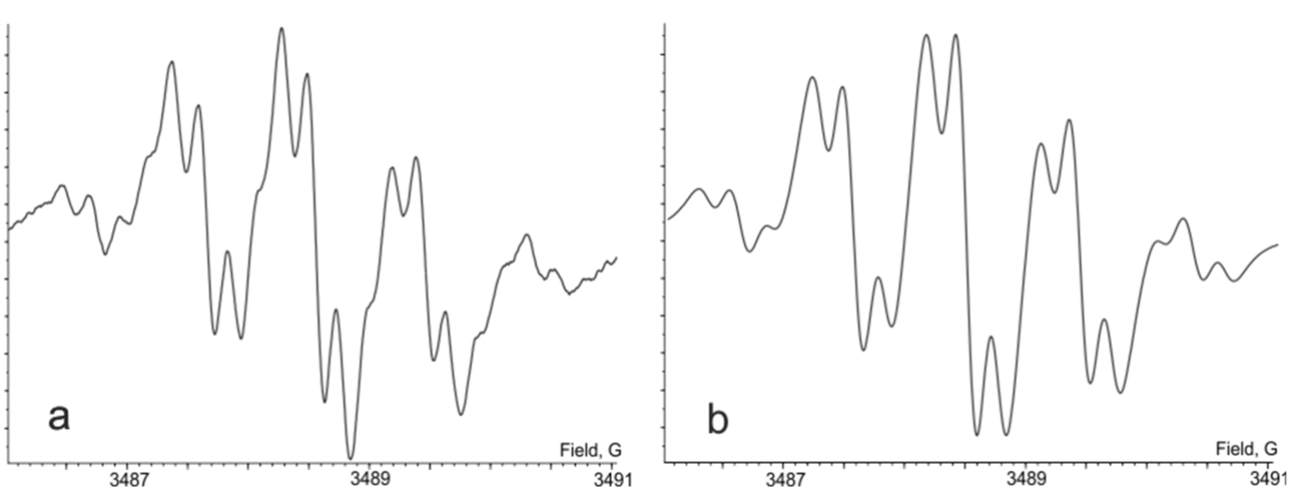
The well-resolved EPR spectrum of **(1^·−^)H_2_** in dimethoxyethane (diluted solution), 273 K: a) experimental and b) simulated.

It is a well-known fact that copper readily dissolves in solutions of *o*-quinones [9–11]. This process is greatly accelerated by the presence of auxiliary ligands, such as phosphines or pyridines. But the oxidation of copper metal with a reduced protonated quinone, which is **(1)H_2_** in fact, was observed for the first time. This process leads to the formation of a paramagnetic product and monitoring by EPR spectroscopy revealed a quintet of triplets signal (Figure 4a and Figure 5a). Upon the addition of a small amount of deuterated water into the reaction mixture, the signal turns into a broadened quintet. This spectrum was assigned to the radical anion species **(1^·−^)H_2_**, which is the product of a one-electron reduction of **(1)H_2_**. The quintet splitting in the EPR spectrum (0.69 G) is explained by the coupling of an unpaired electron with four equivalent protons of the central *p*-phenylene ring. Taking into account the dependence of the spectrum shape on the presence of D_2_O in the solution, we concluded that the triplet pattern (0.19 G) is a result of hyperfine interactions with protons situated at the dioxolene coordination sites. Since the EPR spectrum of **(1)H_2_^·−^** does not exhibit coupling constants on the copper nuclei and considering the symmetrical spin-density distribution in the molecule, we suppose that the copper counterion exists separately form the paramagnetic particle. Tentatively, this counterion is [Cu(dppe)_2_]^+^ and similar tetrahedral species were reported previously [12–14].

It seems useful to find a correlation between the values of the coupling constants on the *p*-phenylene protons in **(1^·^)H_3_** and **(1^·−^)H_2_**, since both species correspond to the trianion oxidation state of the quinone-TTF-quinone core. DFT calculations performed for **(1^.^)H_3_** reveal that the values of the HFS constants for H^1^, H^2^ and H^3^, H^4^ atoms, respectively, should have opposite signs. Considering this, it becomes evident that the averaged value of the coupling constants in **(1^·^)H_3_** (|0.74−2.28|/2 = 0.77) is in good agreement with the value 0.69 G, which was measured for quintet constant in **(1^·−^)H_2_**.

The nature of the phosphine ligand is also important in the reduction process of copper with **(1)H_2_**. The EPR signal corresponding to **(1^·−^)H_2_** could be observed only if a chelating diphosphine was used as an auxiliary ligand in the reaction of copper with **(1)H_2_**. Most likely, phosphines such as dppe, dppb or dppfc provide better stability for the copper(I) ion in solution due to chelation. It should be mentioned, that the reduction of **(1)H_2_** with copper metal proceeds also in the presence of non-bridging phosphines, such as PPh_3_, but the radical anion species **(1^·−^)H_2_** is stable only in the presence of a large counterion such as [Cu(dppe)_2_]^+^.

The signal of the solvent-separated radical ion pair [**(1^·−^)H_2_** Cu(dppe)_2_^+^] was observed in various polar solvents such as DME, THF, methanol, and methylene chloride. We found no significant change in the coupling constants for the EPR spectra recorded in these solvents. We were unable to study the behavior of [**(1^·−^)H_2_** Cu(dppe)_2_^+^] in nonpolar solvents because of the extremely scarce solubility therein. It was also found that the spectrum in THF solution does not display any significant temperature dependence in the range of 180–310 K, except some broadening of the lines below 220 K. This homogeneous broadening is explained by an increase in the viscosity of the solvent. According to these data, **(1^·−^)H_2_** belongs to class III of Robin–Day classification [15] of mixed valence systems. Thus, the molecule does not have distinct catechol or semiquinone oxidation states for the dioxolene moieties on the termini, i.e., an additional electron is symmetrically distributed throughout the ligand. In other words, the *p*-phenylene-extended TTF insertion acts as a conductive bridge, providing fast and effective electronic communication between the dioxolene units.

Remarkably, the symmetric structure of **(1^·−^)H_2_** in solution implies that all four sulfur atoms become equivalent. Due to this fact we can observe the ^33^S (0.75%, S = 3/2, γ_N_ = 2.0557 × 10^3^ rad G^−1^ s^−1^) satellites in the EPR spectrum at high concentration of the radical (Figure 4). The first and the forth components of the sulfur quartet were captured, whereas the central fragment of the satellite signal is shielded by the main spectrum. The low-field component of the ^33^S quartet is well resolved and the HFS pattern arising due to coupling with protons at the *p*-phenylene bridge, is fully reproduced on the satellite spectrum. There is an inhomogeneous broadening of sulfur components: the high-field component of the sulfur quartet appears more broadened than the low-field one. The measured value of the coupling constant on ^33^S is 2.35 G. The hypothesis that this satellite splitting originates from the interaction with the ^13^C nuclei (1.11%, S = 1/2, γ = 6.7283 × 10^4^ rad G^−1^ s^−1^) is untenable, since in this case the value of HFS constant on this carbon should be of 7.06 G. Such a value seems to be too large, because coupling constants which are normally observed for ^13^C–O atoms in semiquinones, are found in the range of 2.5–3.5 G [16–17].

All studied protonated paramagnetic redox forms of quinone **1** display a rather large spin density on the central *p*-phenylene fragment. Such delocalization is accessible if the dihedral angle between the *p*-phenylene and *o*-semiquinone rings is close to zero. This is in a good agreement with the DFT calculation data at the UB3LYP/6-311++G(d,p) level. The geometry optimization gives an almost planar structure for all studied species (see Supporting Information File 1).

It should also be mentioned, that all paramagnetic species were studied in solution only. An isolation of the protonated semiquinones **(1^·^)H** and **(1^·^)H_3_** is unlikely, because these compounds tend to disproportionate leading to diamagnetic products. Unfortunately all attempts to crystallize [**(1^·−^)H_2_** Cu(dppe)_2_^+^] also failed.

## Conclusion

The protonated paramagnetic redox forms of diquinone **1** were investigated by EPR spectroscopy. Since proton-exchange processes between different dioxolene centers are too slow to be observed on the EPR timescale, a picture of spin-density distribution in *o*-quinone-semiquinone **(1^·^)H** and catechol-semiquinone **(1^·^)H_3_** species is determined by the asymmetric structure of the molecular termini. However, a significant portion of the unpaired electron density in these radicals is delocalized outside of the semiquinone ring due to communication via the extended TTF bridge. An identical surrounding of both dioxolene units of the quinone-exTTF-quinone species, which is realized in the case of a solvate separated ion pair, results in fully symmetric delocalization of an unpaired electron on the molecule. The quinone-exTTF-quinone in this form was found to belong to class III of the Robin–Day classification. The coupling constants with ^33^S atoms of dithiol ring were observed.

## Experimental

All reactants were of reagent grade. Solvents were purified by standard methods [18]. X-band EPR spectra were recorded with a Bruker EMX spectrometer. The syntheses of reduced species as well as all spectroscopic investigations were carried out in the absence of oxygen. The standard for *g*-factor was DPPH (*g* = 2.0037). The 2,2’-benzene-1,4-diylbis(6-hydroxy-4,7-di-*tert*-butyl-1,3-benzodithiol-2-ylium-5-olate) **(1^2−^)H_2_** was prepared according to a previously reported procedure [1]. All samples for the EPR study were generated in solution directly before recording of the spectrum.

**(1^·^)H:** 20 mg, (0.03 mmol) of **(1)H_2_** were dissolved in chloroform (10 mL), and degassed. The solution was transferred to an ampoule with an attached EPR tube, containing 0.5 g of PbO_2_. The ampoule was shaken several times and then the solution was poured into the EPR tube.

**(1^.^)H_3_:** The degassed solution of 10 mg (0.015 mmol) of **(1)H_2_** in chloroform (5 mL) was added to an aqueous solution of 100 mg of Na_2_S_2_O_4_ and the mixture was shaken for 10 min. The color of the organic layer turned from deep-violet to red-violet. Then the organic layer was separated, washed with water twice and poured to the EPR ampoule containing a solution of 10 mg of **(1)H_2_** in chloroform (5 mL).

[**(1^·−^)H_2_** Cu(dppe)_2_^+^]: 5 mg (0.007 mmol) of **(1)H_2_** and 30 mg of diphenylphosphinoethane were dissolved in THF (10 mL), then degassed and added to the ampoule with an attached EPR tube, containing 2 g of copper shavings. The ampoule was shaken for 10 min, then the EPR spectrum was recorded.

## Supporting Information

File 1 Additional material.
