# Supplementary material for: Protonated paramagnetic redox forms of di-o-quinone bridged with p-phenylene-extended TTF: A EPR spectroscopy study
Source: Beilstein J Org Chem. 2016 Nov 17;12:2450–6. doi: 10.3762/bjoc.12.238 (PMC5238573; doi:10.3762/bjoc.12.238)
Supplement: File 1 — Additional material. [file Beilstein_J_Org_Chem-12-2450-s001.pdf]

Supporting Information

for

Protonated paramagnetic redox forms of di-*o*-quinone bridged with *p*-phenylene-extended TTF: A EPR spectroscopy study

Nikolay O. Chalkov[1,2], Vladimir K. Cherkasov[1,2], Gleb A. Abakumov[1], Andrey G. Starikov[3] and Viacheslav A. Kuropatov*[1]

Address: [1]Laboratory of Organoelement Compounds, G.A. Razuvaev Institute of Organometallic Chemistry of RAS, 603950, GSP-445, Tropinina str., 49, Nizhny Novgorod, Russia, [2]N. I. Lobachevsky Nizhny Novgorod State University, Gagarina av., 23, Nizhny Novgorod, Russia and [3]Southern Scientific Center of Russian Academy of Science, 344006, Chekhov str., 41, Rostov-on-Don, Russia

Email: Viacheslav A. Kuropatov - viach@iomc.ras.ru

*Corresponding author

Additional material

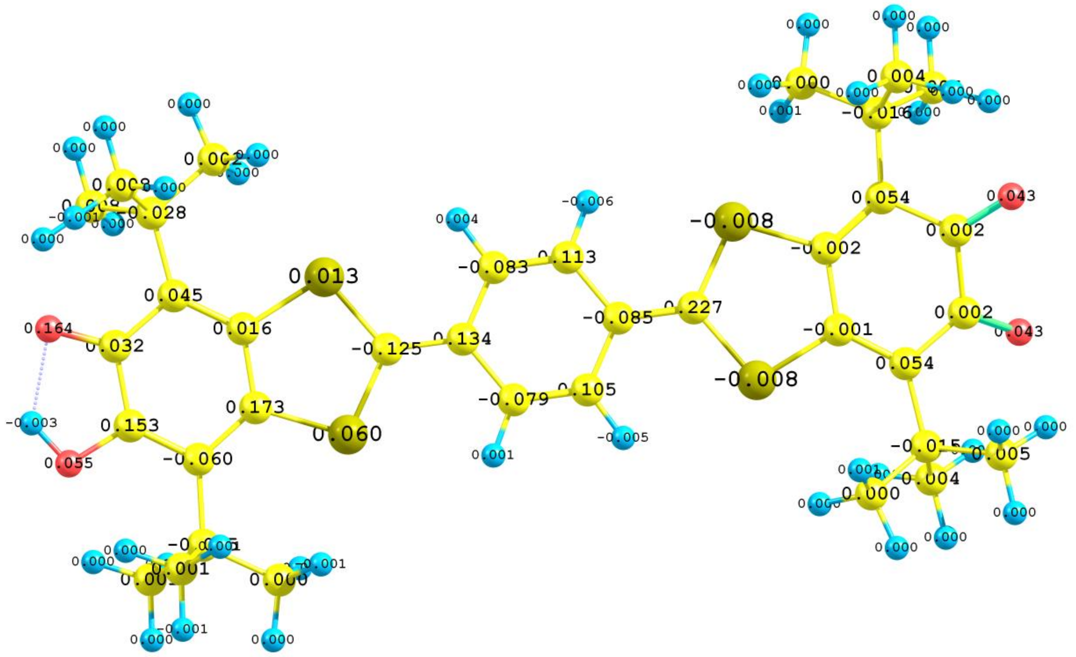

Figure S1: Spin-density distribution for the broken-symmetry state of (**1**$^{\cdot}$)**H**, calculated by UB3LYP/6-311++G(d,p) level of theory.

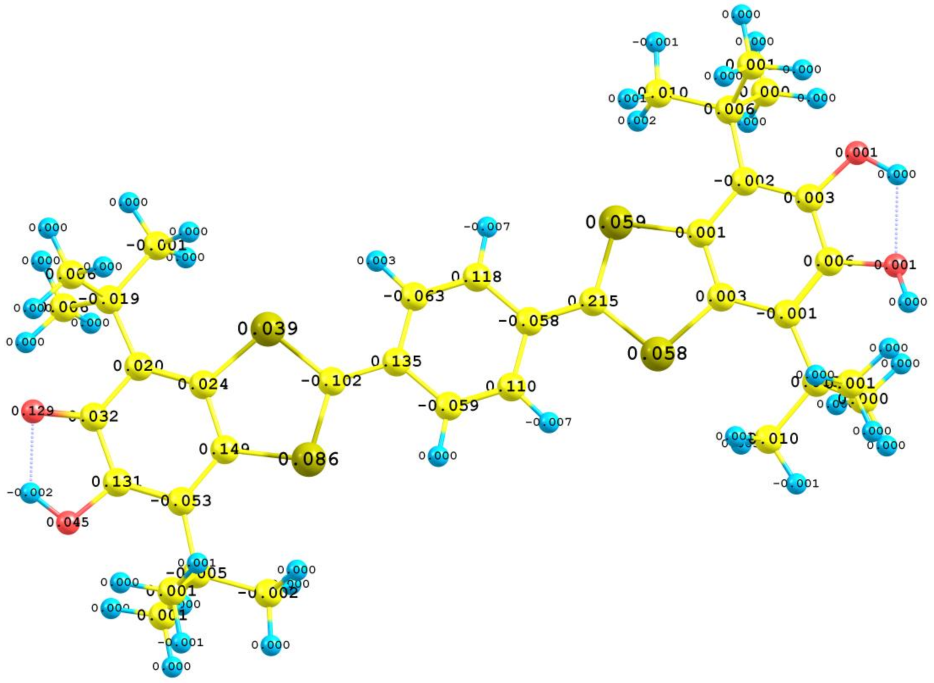

Figure S2: Spin-density distribution for the broken-symmetry state of (**1**$^{\cdot}$)**H$_3$**, calculated by UB3LYP/6-311++G(d,p) level of theory.

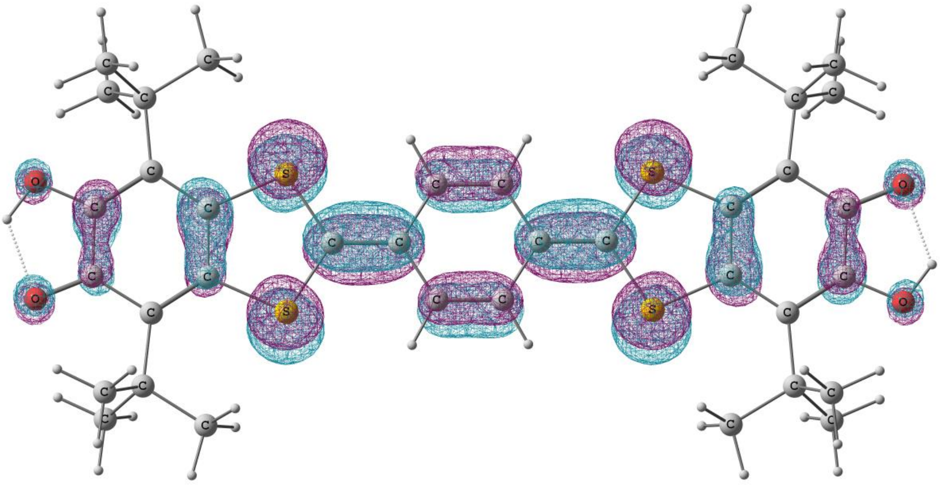

Figure S3: TD DFT B3LYP/6-311++G(d,p) calculated α-SOMO orbital of **(1)H_2** for singlet biradical state.

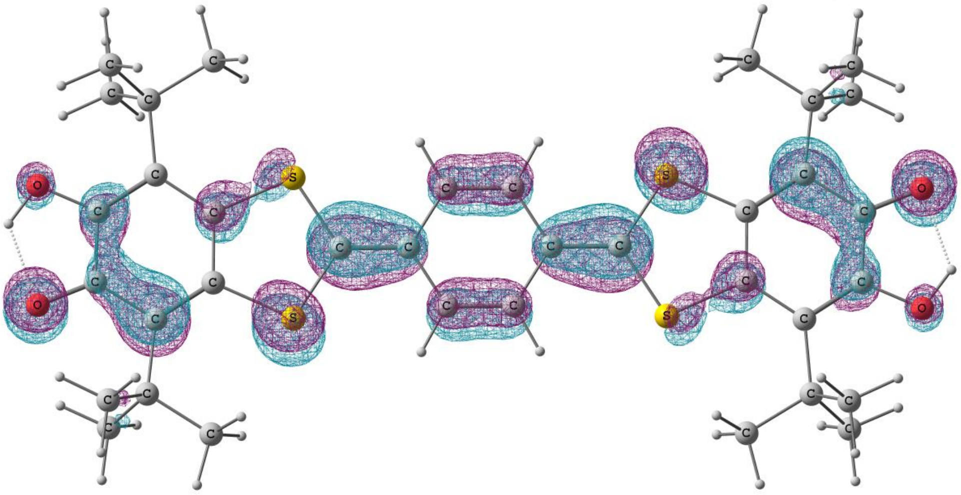

Figure S4: TD DFT B3LYP/6-311++G(d,p) calculated β-SOMO orbital of **(1)H_2** for singlet biradical state.

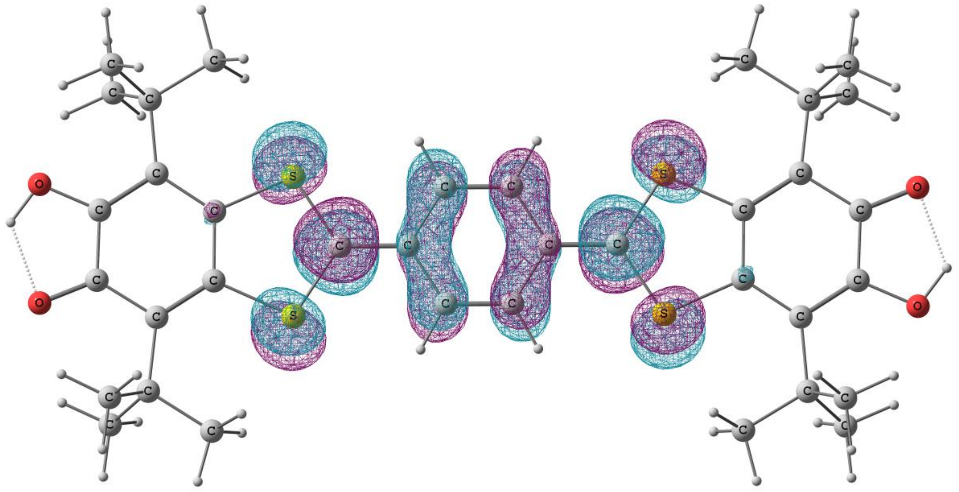

Figure S5: TD DFT B3LYP/6-311++G(d,p) calculated α-LUMO orbital of **(1)H$_2$** for singlet biradical state.

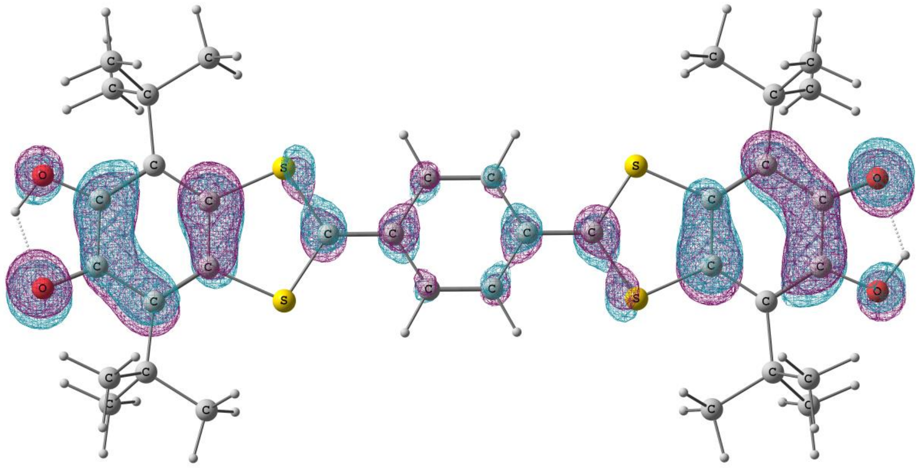

Figure S6: TD DFT B3LYP/6-311++G(d,p) calculated β-LUMO orbital of **(1)H$_2$** for singlet biradical state.
